# Disguised Full-Duplex Covert Communications

**DOI:** 10.3390/s23146515

**Published:** 2023-07-19

**Authors:** Jihwan Moon

**Affiliations:** Department of Mobile Convergence Engineering, Hanbat National University, Daejeon 34158, Republic of Korea; anschino@staff.hanbat.ac.kr

**Keywords:** physical layer security, covert communications, low probability of detection, full duplex, covert rate, detection error probability

## Abstract

Covert communications have arisen as an effective communications security measure that overcomes some of the limitations of cryptography and physical layer security. The main objective is to completely conceal from external devices the very existence of the link for exchanging confidential messages. In this paper, we take a step further and consider a scenario in which a covert communications node disguises itself as another functional entity for even more covertness. To be specific, we study a system where a source node communicates with a seemingly receive-only destination node which, in fact, is full-duplex (FD) and covertly delivers critical messages to another hidden receiver while evading the surveillance. Our aim is to identify the achievable covert rate at the hidden receiver by optimizing the public data rate and the transmit power of the FD destination node subject to the worst-case detection error probability (DEP) of the warden. Closed-form solutions are provided, and we investigate the effects of various system parameters on the covert rate through numerical results, one of which reveals that applying more (less) destination transmit power achieves a higher covert rate when the source transmit power is low (high). Since our work provides a performance guideline from the information-theoretic point of view, we conclude this paper with a discussion on possible future research such as analyses with practical modulations and imperfect channel state information.

## 1. Introduction

Wireless technology has revolutionized the way people live in various ways [1]. However, behind the proliferation of wireless communications are cyberattacks that leave users open to information leakage [2]. To cope with this, cryptography has widely been adopted, which encrypts and decrypts data using secret keys [3]. Nevertheless, this approach has certain limitations, e.g., high complexity for generating secret keys and vulnerability to eavesdroppers with stronger computational power, which are particularly unfavorable for the Internet of Things (IoT) devices. These downsides have led researchers to examine the possibility of utilizing physical layer security [4]. Its main characteristic is that a wireless link from legitimate entities to eavesdroppers can be effectively obstructed, either by nullifying beamforming with multiple antennas, or by disruption with artificial noise (AN) [5]. Hence, the dependency on secret key agreements and the need of avoiding high-powered adversaries can be greatly alleviated.

Still, some applications require an even more strict level of confidentiality. A reconnaissance troop would require reporting its surroundings to the operation center without being detected by enemies in the middle of a military mission [6], or closed networks in security facilities need to make sure that any classified information over the air is concealed from any external party. An adequate technology for such situations is *covert communications* or *low-probability-of-detection* communications that hide the existence of a critical communications link [7].

Covert communications have also been extensively studied for full-duplex (FD) systems. A basic three-node system with a covert transmitter and an FD receiver that simultaneously emits AN was studied in [8]. The authors of [9,10] further treated specific channel properties where only channel distribution information of the transmitter-warden link is available, and where every link is non-coherent with channel distribution information for slow or fast fading, respectively. An antenna selection at the receiver was studied in [11], and a transmission time selection and power control strategy were presented by [12] based on channel state information (CSI). The authors in [13] also verified the effectiveness of a truncated channel inversion power control that ceases covert transmission when the channel of the transmitter–receiver is low. A delay-constrained covert communications with a fixed AN power was investigated in [14], and joint AN power and receiver position optimization problems were discussed in [15,16]. Uncertain locations of a warden node were also taken into consideration in [17]. On top of the AN, a random covert channel selection by the transmitter was studied in [18] to aggravate the confusion of the warden. The maximum detection error probability (DEP) subject to the age of information constraint was identified by [19].

As for more complex FD systems, an FD amplify-and-forward (AF) relay was considered by [20], and the authors in [21] designed an energy harvesting FD decode-and-forward (DF) relay-based covert communications protocol in which the relay forwards and harvests energy simultaneously. Moreover, for integrated satellite–terrestrial communications, an FD relay-aided covert communications from a satellite to a ground node was explored in [22].

For intelligent reflecting surface (IRS)-aided covert communications, a transmit beamforming vector and the reflecting coefficients are jointly optimized when an FD receiver broadcasts random AN to confuse the warden in [23]. An uplink covert communications with the aid of an IRS was also investigated by [24]. Utilizing an active IRS, which is naturally FD, for covert communications between a pair of users was discussed in [25]. The age of information was minimized in [26] when a receiver covertly transmits confidential messages to the transmitter, shielded under public transmissions from the transmitter to the receiver with the aid of an IRS.

For an FD unmanned aerial vehicle (UAV) that collects data from a scheduled user and interferes with unscheduled users with AN, the maximum lowest average covert rate was obtained in [27]. In [28], the location of a covert transmitter UAV was optimized with the help of an FD ground receiver that confuses the warden, and in [29], covert communications with a hovering FD UAV relay assuming Rician air–ground channel was studied. The authors of [30] considered an FD DF UAV relay to aid in covert communications where multiple sensors deliver messages to a remote base station in orthogonal time slots.

Moreover, in a cognitive radio system, covert communications between secondary users in the presence of FD eavesdroppers that interfere with the secondary receiver with AN were examined by [31]. The work in [32] studied covert rate maximization with a half-duplex (HD)/FD mode switching device-to-device (D2D) covert receiver in the presence of an uplink spectrum-sharing cellular network. Both secrecy and covert rates were optimized when an untrusted FD AF relay delivers the covert message to an FD base station that emits AN to the warden in [33].

In the IoT environment, the authors of [34] studied a covert transmitter with an optimized transmission probability, which is wirelessly charged by the AN from an FD receiver. Furthermore, covert uplink transmissions of devices towards FD IoT gateways were optimized based on the mean-field Stackelberg game approach in [35]. With an ambient backscatter system, a radio frequency tag modulates an ambient signal into a covert signal for an FD receiver that concurrently broadcasts AN [36].

Most of past works have assumed that the surveillance nodes have a perfect knowledge of the hardware specifications of covert nodes. However, the covert nodes could disguise themselves as other functional entities for even more covertness. For instance, an original FD node that secretly transmits confidential messages may disguise itself as a receive-only HD node. To the best of the author’s knowledge, there are not enough studies on covert communications that consider such disguised tactics.

In this paper, we study a covert communications system where a source node communicates with a disguised FD destination node. Supposedly receive-only, the destination node covertly delivers critical messages with an invisible extra antenna to another hidden receiver while evading the surveillance of a warden node as much as possible. We identify the optimal public data rate and the transmit power of the FD destination node that maximizes the covert rate at the hidden receiver. Closed-form solutions are provided, and we investigate the effects of various system parameters on the covert rate through numerical results.

Our main contributions are summarized as follows:

- Different from past works which assume that the surveillance party is confident of the hardware specifications of covert nodes, we take a step further and consider a practical scenario in which a covert communications node disguises itself as another functional entity for even more covertness.
- The worst-case DEP is derived in the presence of noise uncertainty at the warden node.
- Noting that covert communications typically suffer from a low data rate due to the stringent DEP requirements, we focus on maximizing the covert rate at the hidden receiver by optimizing the public data rate and the transmit power of the FD destination node subject to the worst-case DEP of the warden.
- We investigate the effects of various system parameters on the covert rate through numerical results, one of which reveals that applying more (less) destination transmit power achieves a higher covert rate when the source transmit power is low (high).
- Since our work provides a performance guideline from the information-theoretic point of view, we suggest analyses with practical modulations and imperfect CSI as valuable future research topics.

## 2. System Model

### 2.1. Received Signals

**Figure 1:**
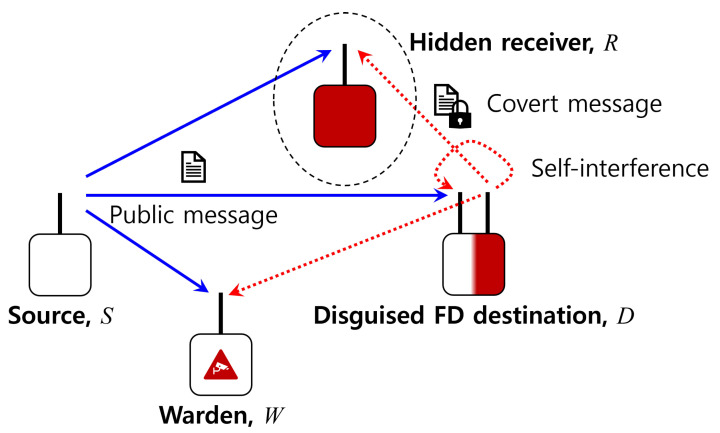
System model.

Figure 1 illustrates the considered system model where the source node *S* sends a public message to the destination node *D*. In the meantime, the seemingly receive-only destination node executes a covert transmission via an invisible extra antenna to the hidden receiver *R* in an FD manner when the warden node *W* keeps monitoring the existence of any such unexpected communications.

First, the received signal at the disguised FD destination node is expressed by

(1) $$\begin{array}{c} y_{D}=h_{SD}\sqrt{P_{S}}x_{P}+{\tilde{h}}_{DD}\sqrt{P_{D}}x_{C}+z_{D}, \end{array}$$

where $h_{\mathrm{XY}}$ stands for the channel coefficient between node X and Y for X,Y∈{S,D,R,W}, ${\tilde{h}}_{DD}∼\mathrm{CN}\left(0,\sigma_{\mathrm{SI}}^{2}\right)$ specifies the residual self-interference channel after self-interference cancellation, $x_{P}∼\mathrm{CN}\left(0,1\right)$ and $x_{C}∼\mathrm{CN}\left(0,1\right)$ denote the public and covert messages, respectively, $P_{S}$ and $P_{D}$ indicate the transmit power at the source node and destination node, respectively, and $z_{X}∼\mathrm{CN}\left(0,\sigma_{X}^{2}\right)$ represents the additive noise at node X. Note that the destination node can keep the CSI of the source node $h_{SD}$ since the covert transmission occurs internally under the normal *S*-*D* communications. The hidden receiver can also easily estimate the CSI of the source and destination nodes, $h_{SR}$ and $h_{DR}$, during channel estimation duration if pilot sequences are informed from the destination node in advance. As for the availability of CSI on the warden node, we assume the worst-case covert communications scenario in this work, meaning that the warden has the perfect knowledge of the CSI from the destination and hidden receiver to identify the worst-case achievable covert rate as a performance guideline in practice.

The source node follows an adaptive transmission policy by which the public data rate $r_{P,D}$ is adjusted based on the feedback from the destination, and the achievable public data rate ${\bar{r}}_{P,D}$ at the destination can be written as [37]

(2) $$\begin{array}{c} {\bar{r}}_{P,D}=\log_{2}\left(1+\frac{{\left|h_{SD}\right|}^{2}P_{S}}{{\left|{\tilde{h}}_{DD}\right|}^{2}P_{D}+\sigma_{D}^{2}}\right). \end{array}$$

The hidden receiver then receives both a public message through the direct link from the source node and a covert message from the destination node as

(3) $$\begin{array}{c} y_{R}=h_{SR}\sqrt{P_{S}}x_{P}+h_{DR}\sqrt{P_{D}}x_{C}+z_{R}. \end{array}$$

Therefore, the hidden receiver needs to successfully decode and eliminate public messages before retrieving covert messages. This implies that the public data rate is also limited by the achievable public data rate ${\bar{r}}_{P,R}$ at the hidden receiver as

(4) $$\begin{array}{c} {\bar{r}}_{P,R}=\log_{2}\left(1+\frac{{\left|h_{SR}\right|}^{2}P_{S}}{{\left|h_{DR}\right|}^{2}P_{D}+\sigma_{R}^{2}}\right). \end{array}$$

The resulting achievable covert rate after removing $x_{P}$ from $y_{R}$ can also be calculated as

(5) $$\begin{array}{c} r_{C,R}=\log_{2}\left(1+\frac{{\left|h_{DR}\right|}^{2}P_{D}}{\sigma_{R}^{2}}\right). \end{array}$$

### 2.2. Covert Message Detection

At the same time, the warden node receives

(6) $$\begin{array}{c} y_{W}=h_{SW}\sqrt{P_{S}}x_{P}+h_{DW}\sqrt{P_{D}}x_{C}+z_{W}. \end{array}$$

It first excludes public messages from $y_{W}$ to obtain the effective residual signal ${\tilde{z}}_{W}≜y_{W}-h_{SW}\sqrt{P_{S}}x_{P}$, assuming it perfectly knows $h_{SW}$ and $P_{S}$ [38]. We then have the following two hypotheses:

(7) $$\begin{array}{c} \begin{array}{cc} H_{0}: & {\tilde{z}}_{W}=z_{W},\\ H_{1}: & {\tilde{z}}_{W}=h_{DW}\sqrt{P_{D}}x_{C}+z_{W}, \end{array} \end{array}$$

where the null hypothesis $H_{0}$ assumes that there does not exist a covert message, and the alternative hypothesis $H_{1}$ presumes that the source node did not send a covert message. This work considers a radiometer [39] as a detection means at the warden, and the sufficient test statistic *T* for (7) after observing N→∞ number of signals reduces to the average residual power $E[{\left|{\tilde{z}}_{W}\right|}^{2}]$ as [40]

(8) $$\begin{array}{c} \begin{array}{cc} H_{0}: & T=\sigma_{W}^{2},\\ H_{1}: & T={\left|h_{DW}\right|}^{2}P_{D}+\sigma_{W}^{2}. \end{array} \end{array}$$

The warden node makes a decision that a covert transmission exists when T≥τ and otherwise when T<τ with some threshold τ.

We consider the uncertainty in the noise variance $\sigma_{W}^{2}$ at the warden node as in [39,41]. Concretely, $\sigma_{W,\mathrm{dB}}^{2}∼U\left({\bar{\sigma }}_{W,\mathrm{dB}}^{2}-\zeta_{\mathrm{dB}},{\bar{\sigma }}_{W,\mathrm{dB}}^{2}+\zeta_{\mathrm{dB}}\right)$ in decibel scale with ${\bar{\sigma }}_{W,\mathrm{dB}}^{2}$ and $\zeta_{\mathrm{dB}}\geq 0$ set to the mean and bounded range, respectively. We then derive the DEP $\mathrm{Pr}\left(e\right)$ that consists of the false alarm and miss probabilities as

(9) $$\begin{array}{c} \mathrm{Pr}\left(e\right)\;\;=\;\;\underset{\mathrm{False}\;\mathrm{alarm}}{\underset{︸}{\mathrm{Pr}\left(T\;\geq \;\tau |H_{0}\right)}}\mathrm{Pr}\left(H_{0}\right)\;+\;\underset{\mathrm{Miss}}{\underset{︸}{\mathrm{Pr}\left(T\;<\;\tau |H_{1}\right)}}\mathrm{Pr}\left(H_{1}\right), \end{array}$$

in which the warden node conjectures that the covert transmission randomly takes place with $\mathrm{Pr}\left(H_{0}\right)=\mathrm{Pr}\left(H_{1}\right)=0.5$ [42]. Making use of the cumulative distribution function (CDF) of $\sigma_{W}^{2}$ (Appendix A),

(10) $$\begin{array}{c} F_{\sigma_{W}^{2}}\left(\nu \right)=\frac{1}{2\ln\zeta }\left(\ln\nu -\ln\frac{{\bar{\sigma }}_{W}^{2}}{\zeta }\right),\nu \in \left[\frac{{\bar{\sigma }}_{W}^{2}}{\zeta },\zeta {\bar{\sigma }}_{W}^{2}\right], \end{array}$$

the false alarm and miss probability are specifically written by

(11) $$\begin{array}{cc} \; & \mathrm{Pr}\left(T\geq \tau |H_{0}\right)=1-F_{\sigma_{W}^{2}}\left(\tau \right),\tau \in T_{1}, \end{array}$$

(12) $$\begin{array}{cc} \; & \mathrm{Pr}\left(T<\tau |H_{1}\right)=F_{\sigma_{W}^{2}}\left(\tau -{\left|h_{DW}\right|}^{2}P_{D}\right),\tau \in T_{2}, \end{array}$$

respectively. Here, $T_{1}≜\left[{\bar{\sigma }}_{W}^{2}/\zeta ,\zeta {\bar{\sigma }}_{W}^{2}\right]$ and $T_{2}≜\left[{\left|h_{DW}\right|}^{2}P_{D}+{\bar{\sigma }}_{W}^{2}/\zeta ,{\left|h_{DW}\right|}^{2}P_{D}+\zeta {\bar{\sigma }}_{W}^{2}\right]$.

We have two different cases depending on the value of ${\left|h_{DW}\right|}^{2}$ and $P_{D}$. If $\zeta {\bar{\sigma }}_{W}^{2}<{\left|h_{DW}\right|}^{2}P_{D}+{\bar{\sigma }}_{W}^{2}/\zeta$,

(13) $$\begin{array}{c} \mathrm{Pr}\left(e\right)=\left\{\begin{array}{cc} \frac{1}{2}\mathrm{Pr}\left(T\geq \tau |H_{0}\right) & \tau \in T_{1},\\ 0 & \tau \in T_{3},\\ \frac{1}{2}\mathrm{Pr}\left(T<\tau |H_{1}\right) & \tau \in T_{2}, \end{array}\right. \end{array}$$

where $T_{3}≜\left[\zeta {\bar{\sigma }}_{W}^{2},{\left|h_{DW}\right|}^{2}P_{D}+{\bar{\sigma }}_{W}^{2}/\zeta \right]$. In contrast, if $\zeta {\bar{\sigma }}_{W}^{2}\geq {\left|h_{DW}\right|}^{2}P_{D}+{\bar{\sigma }}_{W}^{2}/\zeta$,

(14) $$\begin{array}{c} \mathrm{Pr}\left(e\right)\;=\;\;\left\{\;\;\begin{array}{cc} \frac{1}{2}\mathrm{Pr}\left(T\;\geq \;\tau |H_{0}\right),\;\;\; & \;\;\;\tau \;\in \;T_{4},\\ \frac{1}{2}\left(\mathrm{Pr}\left(T\;\geq \;\tau |H_{0}\right)+\mathrm{Pr}\left(T\;<\;\tau |H_{1}\right)\right),\;\;\;\;\; & \;\;\;\tau \;\in \;T_{5},\\ \frac{1}{2}\mathrm{Pr}\left(T\;<\;\tau |H_{1}\right),\;\;\; & \;\;\;\tau \;\in \;T_{6}, \end{array}\right. \end{array}$$

with $T_{4}≜\left[{\bar{\sigma }}_{W}^{2}/\zeta ,{\left|h_{DW}\right|}^{2}P_{D}+{\bar{\sigma }}_{W}^{2}/\zeta \right]$, $T_{5}≜\left[{\left|h_{DW}\right|}^{2}P_{D}+{\bar{\sigma }}_{W}^{2}/\zeta ,\zeta {\bar{\sigma }}_{W}^{2}\right]$ and $T_{6}≜\left[\zeta {\bar{\sigma }}_{W}^{2},{\left|h_{DW}\right|}^{2}P_{D}+\zeta {\bar{\sigma }}_{W}^{2}\right]$.

The warden node may desire a particular τ that can minimize the DEP. To this end, we first see that (11) and (12) are monotonic from 1 to 0 for $\tau \in T_{1}$ and 0 to 1 for $\tau \in T_{2}$, respectively. Moreover, the first derivative of $\mathrm{Pr}\left(T\geq \tau |H_{0}\right)+\mathrm{Pr}\left(T<\tau |H_{1}\right)$ is calculated as

(15) $$\begin{array}{c} \frac{1}{2\ln\zeta }\frac{{\left|h_{DW}\right|}^{2}P_{D}}{\tau \left(\tau -{\left|h_{DW}\right|}^{2}P_{D}\right)}. \end{array}$$

This is always positive for $\tau \in T_{5}$; therefore, the optimal threshold $\tau^{★}$ for the warden node in both (13) and (14) is determined by the boundary between $T_{3}$ and $T_{2}$, or $T_{4}$ and $T_{5}$ as

(16) $$\begin{array}{c} \tau^{★}={\left|h_{DW}\right|}^{2}P_{D}+\frac{1}{\zeta }{\bar{\sigma }}_{W}^{2}. \end{array}$$

Note that (16) provides the worst-case minimum DEP assuming that the warden node knows the exact value of $P_{D}$.

## 3. Problem Formulation

In this work, we aim to identify the optimal public data rate and transmit power of the FD destination node that maximizes the covert rate at the hidden receiver as

(17a) $$\begin{array}{cc} (P1):\; & \max_{P_{D},r_{P}}\;r_{C,R}, \end{array}$$

(17b) $$\begin{array}{cc} \mathrm{subject}\;\mathrm{to}:\; & r_{P}\leq {\bar{r}}_{P,R}, \end{array}$$

(17c) $$\begin{array}{cc} \; & r_{P}\leq {\bar{r}}_{P,D}, \end{array}$$

(17d) $$\begin{array}{cc} \; & r_{P}\geq {\bar{r}}_{P}, \end{array}$$

(17e) $$\begin{array}{cc} \; & {\left.\mathrm{Pr}\left(\mathrm{error}\right)\right|}_{\tau =\tau^{★}}\geq \varepsilon , \end{array}$$

(17f) $$\begin{array}{cc} \; & \zeta {\bar{\sigma }}_{W}^{2}\geq {\left|h_{DW}\right|}^{2}P_{D}+\frac{1}{\zeta }{\bar{\sigma }}_{W}^{2}, \end{array}$$

(17g) $$\begin{array}{cc} \; & 0\leq P_{D}\leq {\bar{P}}_{D}. \end{array}$$

Constraint (17b) guarantees that the hidden receiver successfully decodes and eliminates a public message prior to decoding a covert message, and (17c) indicates the achievable public data rate up to which the destination node can notify the source node to adjust. A minimum quality of service ${\bar{r}}_{P}$ for the public transmission is considered in (17d), and (17e) with (17f) assures the non-zero minimum DEP for 0≤ε≤0.5. Lastly, (17g) shows the power budget ${\bar{P}}_{D}$ at the disguised FD destination node.

## 4. Proposed Solutions

We first note that the covert rate in (17a) is an increasing function of $P_{D}$ while the upper limits of $r_{P}$ in (17b) and (17c) are decreasing functions of $P_{D}$. This means that the covert rate cannot exceed a value at which one of the upper limits becomes $r_{P}$, i.e., $r_{P}=\min\left({\bar{r}}_{P,R},{\bar{r}}_{P,D}\right)$. Therefore, it is optimal for $r_{P}$ to be as low as possible for the maximum covert rate as

(18) $$\begin{array}{c} r_{P}^{★}={\bar{r}}_{P}. \end{array}$$

We now simplify (P1) using the monotonicity of logarithms as

(19a) $$\begin{array}{cc} (P1.1):\; & \max_{P_{D}}\;P_{D}, \end{array}$$

(19b) $$\begin{array}{cc} \mathrm{subject}\;\mathrm{to}:\; & P_{D}\leq \frac{1}{{\left|h_{DR}\right|}^{2}}\left(\frac{{\left|h_{SR}\right|}^{2}P_{S}}{2^{{\bar{r}}_{P}}-1}-\sigma_{R}^{2}\right), \end{array}$$

(19c) $$\begin{array}{cc} \; & P_{D}\leq \frac{1}{{\left|{\tilde{h}}_{DD}\right|}^{2}}\left(\frac{{\left|h_{SD}\right|}^{2}P_{S}}{2^{{\bar{r}}_{P}}-1}-\sigma_{D}^{2}\right), \end{array}$$

(19d) $$\begin{array}{cc} \; & P_{D}\leq \left(\zeta^{\left(1-4\varepsilon \right)}-\frac{1}{\zeta }\right)\frac{{\bar{\sigma }}_{W}^{2}}{{\left|h_{DW}\right|}^{2}}, \end{array}$$

(19e) $$\begin{array}{cc} \; & P_{D}\leq \left(\zeta -\frac{1}{\zeta }\right)\frac{{\bar{\sigma }}_{W}^{2}}{{\left|h_{DW}\right|}^{2}}, \end{array}$$

(19f) $$\begin{array}{cc} \; & 0\leq P_{D}\leq {\bar{P}}_{D}. \end{array}$$

The right-hand side of (19d) is larger than or equal to that of (19e) for 0≤ε≤0.5, implying that satisfying (19d) automatically fulfills (19e). Therefore, the optimal transmit power can be obtained by taking the minimum of the upper bounds from (19b)–(19d) and (19f) as

(20) $$\begin{array}{cc} P_{D}^{★}\;\;=\;\;\min\; & \left\{\;\frac{1}{{\left|h_{DR}\right|}^{2}}\left(\frac{{\left|h_{SR}\right|}^{2}P_{S}}{2^{{\bar{r}}_{P}}-1}\;-\;\sigma_{R}^{2}\right),\frac{1}{{\left|{\tilde{h}}_{DD}\right|}^{2}}\left(\frac{{\left|h_{SD}\right|}^{2}P_{S}}{2^{{\bar{r}}_{P}}-1}\;-\;\sigma_{D}^{2}\right),\left(\zeta^{\left(1-4\varepsilon \right)}\;-\;\frac{1}{\zeta }\right)\frac{{\bar{\sigma }}_{W}^{2}}{{\left|h_{DW}\right|}^{2}},{\bar{P}}_{D}\right\}. \end{array}$$

**Remark 1.**  *When the D-R link is extremely strong, i.e., ${\left|h_{DR}\right|}^{2}\to \infty$, we have $P_{D}^{★}\to 0$ since the hidden receiver cannot eliminate a source message in the presence of a dominant covert message, which is a prerequisite. When there exists excessive FD self-interference, i.e., ${\left|{\tilde{h}}_{DD}\right|}^{2}\to \infty$, we also have $P_{D}^{★}\to 0$ for the public data rate, which cannot reach the given threshold ${\bar{r}}_{P}$. Lastly, when the channel gain of the D-W link is exceptionally high, i.e., ${\left|h_{DW}\right|}^{2}\to \infty$, we have $P_{D}^{★}\to 0$ as well, since the absence and existence of covert transmission will cause a large difference in the received power at the warden, making it easier to detect any covert transmissions.*

## 5. Numerical Results

We evaluate the proposed solutions for covert communications with the disguised FD node through numerical results. The effects of various system parameters such as the source transmit power, disguised FD destination transmit power budget, noise uncertainty bound, and minimum DEP threshold on the achievable covert rate $r_{C,R}$ in (5) with the derived optimal destination transmit power $P_{D}^{★}$ in (20) are examined in the upcoming figures. We also focus on verification that $P_{D}^{★}$ fulfills the DEP requirements for any desired threshold ε in (17e) and compare with baseline schemes to stress the significance of $P_{D}^{★}$.

**Figure 2:**
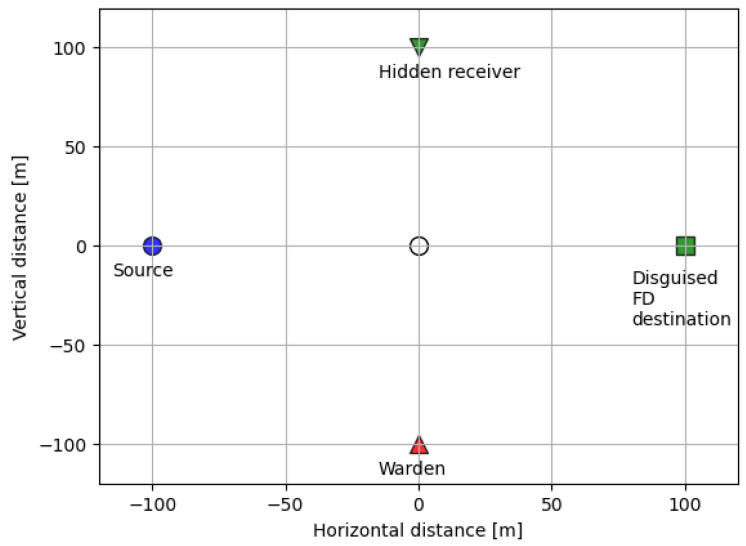
Node placements.

The distance-dependent channel model is adopted for $h_{\mathrm{XY}}$ [43]. Concretely, we let ${\left|h_{\mathrm{XY}}\right|}^{2}=L_{\mathrm{XY}}{\left|{\hat{h}}_{\mathrm{XY}}\right|}^{2}$, where $L_{\mathrm{XY}}≜L_{0}{\left(d_{\mathrm{XY}}/d_{0}\right)}^{-\beta }$ indicates the path loss between X and Y. Here, $L_{0}$ stands for the path loss at a reference distance $d_{0}=1$ m, β represents the path loss exponent, and $d_{\mathrm{XY}}$ indicates the distance between X and Y. Also, the small-scale channel variable ${\hat{h}}_{\mathrm{XY}}$ follows CN(0,1). The four nodes are placed with certain distances from the origin O=(0,0) in a Cartesian coordinate system such that the coordinates of *S*, *D*, *R*, *W* are $\left(-d_{OS},0\right)$, $\left(d_{OD},0\right)$, $\left(0,d_{OR}\right)$, $\left(0,-d_{OW}\right)$, respectively (Figure 2). The overall system parameters are fixed as follows unless otherwise stated: the bandwidth B=20 MHz, $d_{OX}=100$ m, source transmit power $P_{S}=23$ dBm, destination transmit power budget ${\bar{P}}_{D}=23$ dBm, public message quality of service ${\bar{r}}_{P,D}=0.1$ bps/Hz, mean noise power at the warden node ${\bar{\sigma }}_{W}^{2}=-160$ dBm/Hz, noise uncertainty bound ζ=5 dB, noise power at the destination node and hidden receiver $\sigma_{D}^{2}=\sigma_{R}^{2}=-160$ dBm/Hz, residual self-interference $\sigma_{\mathrm{SI}}^{2}=-100$ dB, minimum DEP threshold ε=0.45, and pathloss exponent β=3.5.

**Figure 3:**
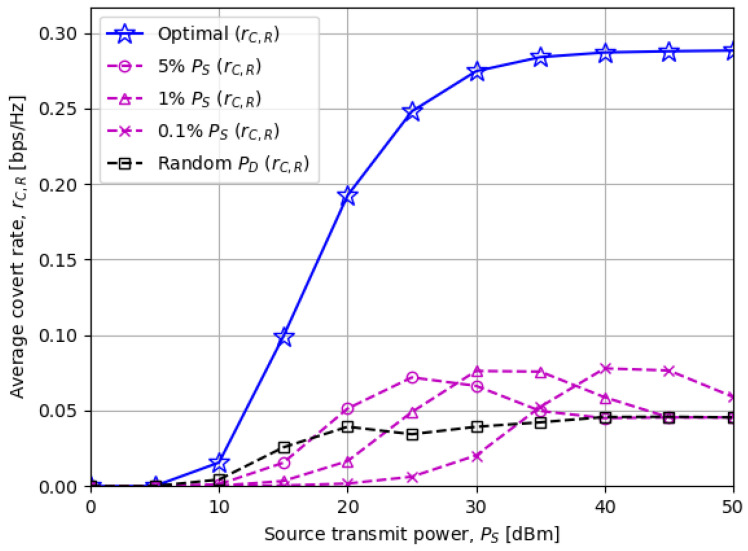
The average covert rate $r_{C,R}$ versus the source node power $P_{S}$: Applying more (less) $P_{D}$ is preferred when $P_{S}$ is low (high).

Figure 3 shows the average covert rate $r_{C,R}$ as the source transmit power $P_{S}$ varies. Motivated by the fact that the destination transmit power $P_{D}$ should be much lower than $P_{S}$ for successful covert transmissions, we compare the optimal scheme with “α% $P_{S}$” in which $P_{D}$ is fixed as $\min(\alpha \%\;\mathrm{of}\;P_{S},{\bar{P}}_{D})$. We notice that the proposed public data rate in (18) and destination transmit power in (20) result in the highest covert rate for every $P_{S}$ value, indicating the importance of optimizing $r_{P}$ and $P_{D}$.

We also observe from the “α% $P_{S}$” schemes that applying more $P_{D}$ to a covert transmission induces a higher covert rate when $P_{S}$ is low, while less $P_{D}$ is preferred when $P_{S}$ is high. First, when $P_{S}$ is low, the public data rate constraints in (17b) and (17c) dominate determining $P_{D}^{★}$ from (20). If $\nu ≜\min(E\left[{\left|h_{SR}\right|}^{2}/\left({\left|h_{DR}\right|}^{2}\left(2^{{\bar{r}}_{P}}-1\right)\right)\right],E\left[{\left|h_{SD}\right|}^{2}/\left({\left|{\tilde{h}}_{DD}\right|}^{2}\left(2^{{\bar{r}}_{P}}-1\right)\right)\right])$, then any “α% $P_{S}$” schemes with α%>ν are likely to be infeasible on average. It can be inferred from Figure 3 that that ν≥5% for our system setup since “5% $P_{S}$” performs the best among the other fixed $P_{D}$ schemes.

On the other hand, when $P_{S}$ is high, the DEP constraint in (17e) and the power budget ${\bar{P}}_{D}$ dominate deciding $P_{D}^{★}$. Hence, only the “α% $P_{S}$” schemes with sufficiently low α% can meet these requirements and be feasible on average. This explains the reason why “0.1% $P_{S}$” outperforms those with higher α% in Figure 3 in the high $P_{S}$ region.

**Figure 4:**
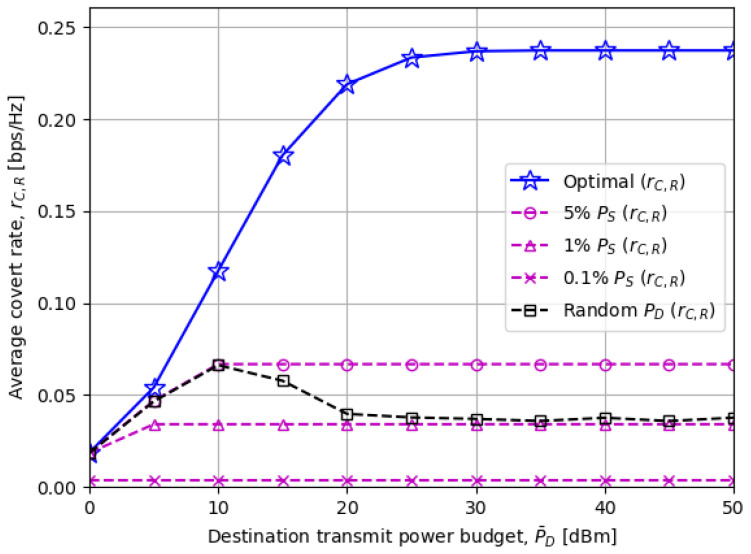
The average covert rate $r_{C,R}$ versus the destination node power budget ${\bar{P}}_{D}$: Close performance among the presented schemes for low ${\bar{P}}_{D}$ since the optimal $P_{D}$ is dominantly limited by ${\bar{P}}_{D}$.

Figure 4 compares the average covert rate $r_{C,R}$ as the destination transmit power budget ${\bar{P}}_{D}$ changes. It can be seen that “5% $P_{S}$” and random $P_{D}$ schemes perform close to the optimal scheme when ${\bar{P}}_{D}$ is low. The reasons are that $P_{D}^{★}$ is dominated by ${\bar{P}}_{D}$ in this region and that fixed or randomly chosen $P_{D}$ in the compared schemes is reduced to ${\bar{P}}_{D}$ if $P_{D}>{\bar{P}}_{D}$. For the rest of the ${\bar{P}}_{D}$ regions, our proposed solutions achieve the highest covert rate which once more highlights the necessity of optimizing the $r_{P}$ and $P_{D}$.

**Figure 5:**
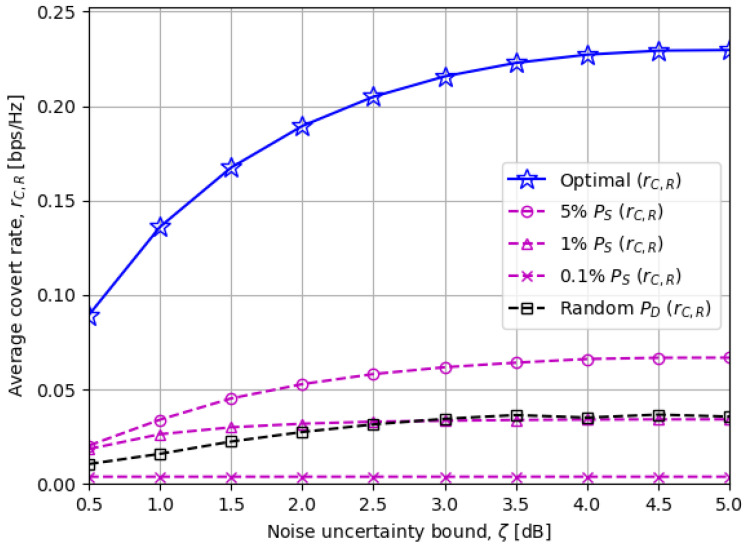
The average covert rate $r_{C,R}$ versus the noise uncertainty bound ζ [dB]: The more unsettled ζ is, the easier it is to perform covert transmissions.

Figure 5 demonstrates the average covert rate $r_{C,R}$ for different noise uncertainty bounds ζ at the warden node. Quantitatively, a larger ζ widens the upper bound for $P_{D}^{★}$ in (20) so that the covert rate, which is proportional to $P_{D}$, has more chance to be enhanced on average. Also, from the qualitative aspect, the larger ζ there is, the more confusion the warden node undergoes in deciding the existence of covert communications.

**Figure 6:**
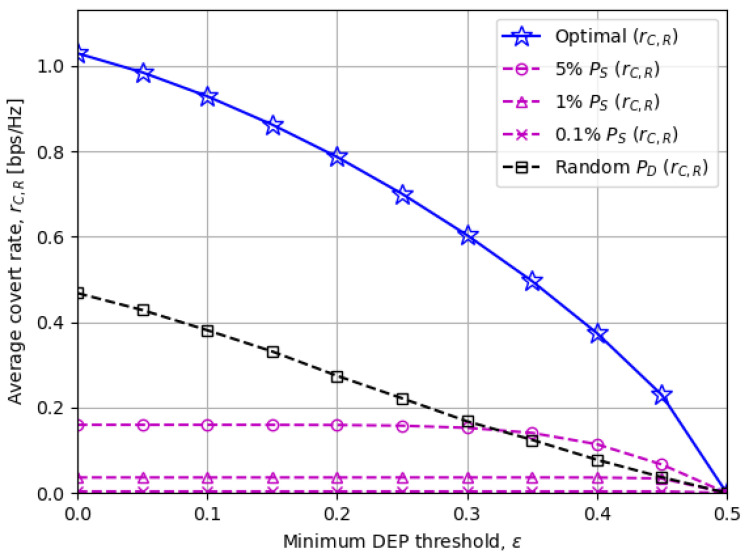
The average covert rate $r_{C,R}$ versus the minimum DEP threshold ε.

**Figure 7:**
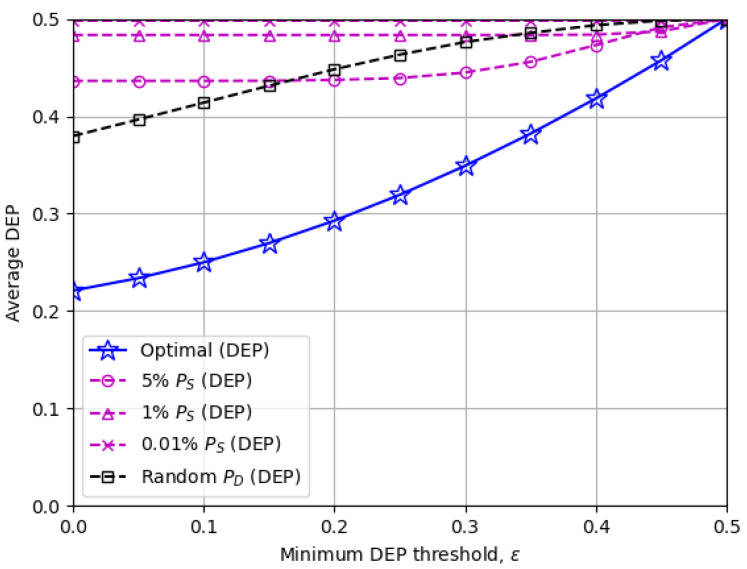
The average DEP versus the minimum DEP threshold ε: The optimal scheme benefits from the best trade-off between the covert rate and DEP for a given ε.

Figure 6 and Figure 7 illustrate the average covert rate $r_{C,R}$ and DEP, respectively, when the minimum DEP threshold ε changes. The covert rates decline in a monotonic manner as ε increases and eventually become zero when a perfect DEP of 0.5 is imposed on. Figure 7 also verifies that the proposed optimal solutions provide just enough DEP above the threshold ε in general, while the other baseline schemes achieve unnecessarily high DEP by sacrificing the covert rate.

## 6. Discussion

### 6.1. Performance

The numerical results from Figure 3, Figure 4, Figure 5, Figure 6 and Figure 7 confirmed that the optimized transmit power at the disguised FD destination node has a significant impact on the covert rate performance. Furthermore, Figure 3 revealed an interesting relationship between the source and destination transmit power, which is that applying more (less) destination transmit power achieves a higher covert rate when the source transmit power is low (high). Figure 7 also showed that the optimal destination transmits power exploits the best trade-off between the covert rate and minimum DEP threshold.

### 6.2. Applications

The considered system model and obtained solution may be used in various practical networks. In the military, a reconnaissance troop may place on the adversary side a counterfeit FD device that periodically reports situations while disguising itself as a typical half-duplex one. Moreover, the authors in [44] demonstrated the feasibility of FD on a low Earth orbit (LEO) satellite, and the future non-terrestrial military network would utilize the disguise tactic proposed in this paper for either defense or offense purposes. For surveillance, an IoT network administrator may exploit a disguised FD node to covertly monitor any suspicious users that misuse the network for prohibitive activities.

## 7. Conclusions

In this paper, we studied a covert communications system where a source node communicates with a disguised FD destination node. Supposedly receive-only, the destination node covertly delivers critical messages to another hidden receiver while evading the surveillance of a warden node as much as possible. We identified the optimal public data rate and the transmit power of the FD destination node that maximizes the covert rate at the hidden receiver.

The obtained closed-form solution exhibited the following: When the destination–receiver link is extremely strong, the optimal destination transmit power approaches zero since the hidden receiver cannot eliminate a source message prior to retrieving a covert message. If the self-interference is not sufficiently suppressed, the optimal destination transmit power approaches zero in this case as well since the public data rate cannot reach the required quality of service. In addition, when the destination–warden channel gain is exceptionally high, the optimal destination transmit power approaches zero since the large difference in the received power at the warden makes it easier to detect the covert link.

The extensive numerical results presented a phenomenon that applying more (less) destination transmit power achieves a higher covert rate when the source transmit power is low (high). It was also verified that the optimal destination transmits power yields the best balance between the covert rate and minimum DEP threshold.

Since our work provides a performance guideline from the information-theoretic point of view, we suggest analyses with practical modulations and imperfect CSI as valuable future research topics.

## Data Availability

Not applicable.

## Appendix A

First note that $\sigma_{W,\mathrm{dB}}^{2}=10\log_{10}\sigma_{W}^{2}$. By the transformation of probability density functions (PDFs) $f_{\sigma_{W}^{2}}\left(\nu \right)=\left|J\right|f_{\sigma_{W,\mathrm{dB}}^{2}}\left(\nu \right)$ with the Jacobian *J* defined by [45]

(A1) $$\begin{array}{c} J=\frac{\partial \sigma_{W,\mathrm{dB}}^{2}}{\partial \sigma_{W}^{2}}=\frac{10}{\sigma_{W}^{2}\ln10}, \end{array}$$

the PDF of $\sigma_{W}^{2}$ becomes

(A2) $$\begin{array}{c} f_{\sigma_{W}^{2}}\left(\nu \right)=\frac{10}{\nu \ln10}\cdot \frac{1}{2\zeta_{\mathrm{dB}}}=\frac{10}{\nu \ln10}\cdot \frac{1}{2\cdot 10\log_{10}\zeta }=\frac{1}{2\ln\zeta \cdot \nu }. \end{array}$$

Accordingly, the CDF of $\sigma_{W}^{2}$ is obtained by

(A3) $$\begin{array}{c} F_{\sigma_{W}^{2}}\left(\nu \right)=\int_{\frac{{\bar{\sigma }}_{W}^{2}}{\zeta }}^{\nu }f_{\sigma_{W}^{2}}\left(\nu \right)d\nu , \end{array}$$

which yields (10).

## References

[1] Qazi S., Khawaja B.A., Farooq Q.U. (2022). IoT-Equipped and AI-Enabled Next Generation Smart Agriculture: A Critical Review, Current Challenges and Future Trends. IEEE Access.

[2] Zhang J., Yan Z., Fei S., Wang M., Li T., Wang H. (2022). Is Today’s End-to-End Communication Security Enough for 5G and Its Beyond?. IEEE Netw..

[3] Forouzan B.A. (2007). Cryptography and Network Security.

[4] Wyner A.D. (1975). The Wire-Tap Channel. Bell Syst. Tech. J..

[5] Angueira P., Val I., Montalbán J., Seijo Ó., Iradier E., Fontaneda P.S., Fanari L., Arriola A. (2022). A Survey of Physical Layer Techniques for Secure Wireless Communications in Industry. IEEE Commun. Surv. Tutor..

[6] Jiang X., Chen X., Tang J., Zhao N., Zhang X.Y., Niyato D., Wong K.-K. (2021). Covert Communication in UAV-Assisted Air-Ground Networks. IEEE Wirel. Commun..

[7] Bash B.A., Goeckel D., Towsley D., Guha S. (2015). Hiding information in noise: Fundamental limits of covert wireless communication. IEEE Commun. Mag..

[8] Shahzad K., Zhou X., Yan S., Hu J., Shu F., Li J. (2018). Achieving Covert Wireless Communications Using a Full-Duplex Receiver. IEEE Trans. Wirel. Commun..

[9] Xu T., Xu L., Liu X., Lu Z. Covert Communication with A Full-Duplex Receiver Based on Channel Distribution Information. Proceedings of the 2018 12th International Symposium on Antennas, Propagation and EM Theory (ISAPE).

[10] Zheng M., Hamilton A., Ling C. (2021). Covert Communications with a Full-Duplex Receiver in Non-Coherent Rayleigh Fading. IEEE Trans. Commun..

[11] Yang L., Yang W., Xu S., Tang L., He Z. Achieving Covert Wireless Communications Using a Full-Duplex Multi-Antenna Receiver. Proceedings of the 2019 IEEE 5th International Conference on Computer and Communications (ICCC).

[12] Wang J., Li Y., Tang W., Li X., Li S. Channel State Information Based Optimal Strategy for Covert Communication. Proceedings of the 2019 11th International Conference on Wireless Communications and Signal Processing (WCSP).

[13] Hu J., Yan S., Zhou X., Shu F., Li J. (2019). Covert Wireless Communications With Channel Inversion Power Control in Rayleigh Fading. IEEE Trans. Veh. Technol..

[14] Shu F., Xu T., Hu J., Yan S. (2019). Delay-Constrained Covert Communications With a Full-Duplex Receiver. IEEE Wirel. Commun. Lett..

[15] Zhao Y., Li Z., Cheng N., Wang D., Quan W., Shen X. Joint Power and Position Optimization for the Full-Duplex Receiver in Covert Communication. Proceedings of the ICC 2020-2020 IEEE International Conference on Communications (ICC).

[16] Xu R., Guan L., Zhao Y., Li Z., Wang D. Robust Power and Position Optimization for the Full-Duplex Receiver in Covert Communication. Proceedings of the 2021 IEEE Global Communications Conference (GLOBECOM).

[17] Chen X., Sun W., Xing C., Zhao N., Chen Y., Yu F.R., Nallanathan A. (2021). Multi-Antenna Covert Communication via Full-Duplex Jamming Against a Warden With Uncertain Locations. IEEE Trans. Wirel. Commun..

[18] Che B., Yang W., Lu X. Covert Communication for Multi-Channel Transmission with A Full-Duplex Receiver. Proceedings of the 2021 13th International Conference on Wireless Communications and Signal Processing (WCSP).

[19] Wang Y., Yan S., Yang W., Cai Y. (2021). Covert Communications With Constrained Age of Information. IEEE Wirel. Commun. Lett..

[20] Sun R., Yang B., Ma S., Shen Y., Jiang X. (2021). Covert Rate Maximization in Wireless Full-Duplex Relaying Systems With Power Control. IEEE Trans. Commun..

[21] Li Y., Zhao R., Deng Y., Shu F., Nie Z., Aghvami A.H. (2020). Harvest-and-Opportunistically-Relay: Analyses on Transmission Outage and Covertness. IEEE Trans. Wirel. Commun..

[22] Wu Z., Guo K., Zhu S. (2023). Covert Communication for Integrated Satellite–Terrestrial Relay Networks with Cooperative Jamming. Electronics.

[23] Wang C., Li Z., Shi J., Ng D.W.K. (2021). Intelligent Reflecting Surface-Assisted Multi-Antenna Covert Communications: Joint Active and Passive Beamforming Optimization. IEEE Trans. Commun..

[24] Pejoski S., Hadzi-Velkov Z., Zlatanov N. (2022). Full-Duplex Covert Communications Assisted by Intelligent Reflective Surfaces. IEEE Commun. Lett..

[25] Wang M., Xu Z., Xia B., Guo Y. (2023). Active Intelligent Reflecting Surface Assisted Covert Communications. IEEE Trans. Veh. Technol..

[26] Wang C., Li Z., Zheng T.-X., Ng D.W.K., Al-Dhahir N. (2023). Intelligent Reflecting Surface-Aided Full-Duplex Covert Communications: Information Freshness Optimization. IEEE Trans. Wirel. Commun..

[27] Zhou X., Yan S., Shu F., Chen R., Li J. (2021). UAV-Enabled Covert Wireless Data Collection. IEEE J. Sel. Areas Commun..

[28] Guo Z., Zhao S., Wang J., Lit H., Shen Y. Optimal Location Design for UAV Covert Communications with a Full-Duplex Receiver. Proceedings of the 2022 International Conference on Networking and Network Applications (NaNA).

[29] Zhang R., Chen X., Liu M., Zhao N., Wang X., Nallanathan A. (2022). UAV Relay Assisted Cooperative Jamming for Covert Communications Over Rician Fading. IEEE Trans. Veh. Technol..

[30] Li M., Tao X., Wu H., Li N. (2023). Joint Trajectory and Resource Optimization for Covert Communication in UAV-Enabled Relaying Systems. IEEE Trans. Veh. Technol..

[31] Yang J., Zhou H., Chen R., Shi J., Li Z. Covert Communication Against a Full-Duplex Adversary in Cognitive Radio Networks. Proceedings of the GLOBECOM 2022-2022 IEEE Global Communications Conference.

[32] Yang Y., Yang B., Shen S., She Y., Taleb T. (2023). Covert Rate Study for Full-Duplex D2D Communications Underlaid Cellular Networks. IEEE Internet Things J..

[33] Sun R., Yang B., Shen Y., Jiang X., Taleb T. (2023). Covertness and Secrecy Study in Untrusted Relay-Assisted D2D Networks. IEEE Internet Things J..

[34] Wang Y., Yan S., Yang W., Zhong C., Ng D.W.K. (2022). Probabilistic Accumulate-Then-Transmit in Wireless-Powered Covert Communications. IEEE Trans. Wirel. Commun..

[35] Feng S., Lu X., Sun S., Niyato D. (2022). Mean-Field Artificial Noise Assistance and Uplink Power Control in Covert IoT Systems. IEEE Trans. Wirel. Commun..

[36] Liu J., Yu J., Chen X., Zhang R., Wang S., An J. (2022). Covert Communication in Ambient Backscatter Systems With Uncontrollable RF Source. IEEE Trans. Commun..

[37] Cover T.M., Thomas J.A. (2005). Elements of Information Theory.

[38] Kim S.W., Ta H.Q. (2022). Covert Communications Over Multiple Overt Channels. IEEE Trans. Commun..

[39] He B., Yan S., Zhou X., Lau V.K.N. (2017). On Covert Communication With Noise Uncertainty. IEEE Commun. Lett..

[40] Sobers T.V., Bash B.A., Guha S., Towsley D., Goeckel D. (2017). Covert Communication in the Presence of an Uninformed Jammer. IEEE Trans. Wirel. Commun..

[41] Si J., Li Z., Zhao Y., Cheng J., Guan L., Shi J., Al-Dhahir N. (2021). Covert Transmission Assisted by Intelligent Reflecting Surface. IEEE Trans. Commun..

[42] Liu Z., Liu J., Zeng Y., Ma J. (2018). Covert Wireless Communications in IoT Systems: Hiding Information in Interference. IEEE Wirel. Commun..

[43] Moon J., Lee S.H., Lee H., Lee I. (2019). Proactive Eavesdropping With Jamming and Eavesdropping Mode Selection. IEEE Trans. Wirel. Commun..

[44] Grayver E., Keating R., Parower A. Feasibility of full duplex communications for LEO satellite. Proceedings of the 2015 IEEE Aerospace Conference.

[45] Ross S. (2019). A First Course in Probability.

